# Oral health-related quality of life and dental occlusion before and after alveolar and hard palate reconstruction at the time of mixed dentition

**DOI:** 10.3389/froh.2025.1669110

**Published:** 2025-11-17

**Authors:** Hilda González-Olivares, Kathrine Jáuregui-Renaud

**Affiliations:** 1 UMAE Hospital de Pediatría, Centro Médico Nacional Siglo XXI, Instituto Mexicano del Seguro Social, Ciudad de México, México; 2 Unidad de Investigación Médica en Otoneurología, Instituto Mexicano del Seguro Social, Ciudad de México, México

**Keywords:** cleft palate, dental occlusion, oral-health related quality of life, mixed dentition, nasoalveolar fistula

## Abstract

**Aim:**

In children 8–10 years old with not syndromic cleft lip and palate (nsCLP) and intentionally unrepaired nasoalveolar fistula, to assess the difference in oral health-related quality of life before and 6 months after the reconstruction, compared to age matched children with no birth abnormalities, considering dental occlusion.

**Methods:**

52 children participated in the study, 26 with nsCLP and 26 with no birth abnormalities. At inclusion in the study, dental occlusion was assessed by the Angle classification and the Dental Aesthetic Index in all the participants, and also by the GOSLON yardstick index in participants with nsCLP. Oral health-related quality of life was evaluated by the Child Perceptions Questionnaire (CPQ_8-10_) twice, with 6 months in between. Bivariate and repeated measures multivariate analyses were performed with *p* ≤ 0.05.

**Results:**

In participants with/ without nsCLP, oral-health related quality of life was significantly related to dental occlusion and to age. In participants with nsCLP, after reconstruction, quality of life improved mainly on the emotional and social well-being domains, with influence and interaction between age and dental occlusion.

**Comment:**

In children with nsCLP and intentionally unrepaired nasoalveolar fistula, the earliest possible repair could be beneficial for their emotional and social well-being. To start prompt orthopaedic treatment, early evaluation of dental occlusion should be promoted in both children with and without nsCLP.

## 1 Introduction

Cleft lip and palate (CLP) are among the most prevalent congenital craniofacial malformations. They are caused by complex genetic and environmental factors and comprise a variety of defects, which can develop as a syndrome or as a single abnormality (not syndromic CLP, nsCLP). Orofacial clefts can be unilateral or bilateral, include the lip with/without the palate or just the palate (complete or incomplete) and affect the primary and/or the secondary palate (submucous or overt) (1). The metanalysis of 55 international studies (*n* = 17,894,673) showed a prevalence of cleft lip and palate of 0.45/1,000 live births [95% C.I. (Confidence Interval) 0.38–0.52] (2).

The disturbed facial growth and dental development implicate aesthetic, functional and psychosocial consequences (3, 4). The surgical repair is fundamental for adequate facial growth, dentition development and speech formation. However, the phenotypic variants entail a diversity of reconstruction procedures. To close the palatal cleft, the two main options are a one stage repair of both the soft and the hard palate, or a two-stage repair, when the soft palate is repaired first and the hard palate closure is delayed either at infancy or until mixed dentition [for review see (5)]. To achieve the alveolar and hard palate reconstruction at the time of mixed dentition, iliac crest bone grafting is performed (6). This second phase at the age of mixed dentition is favourable for maxillary growth, with better dental arch relationships (7).

Patients with CLP have to overcome speech, hearing, nutrition, mental and social challenges. Interdisciplinary treatment can lead to improvement in all domains (8), including self-esteem, self-confidence, and social competence (9). A scoping review on the quality of life of children with CLP, showed the positive effects of multidisciplinary care, with favourable quality of life scores; however, inconsistent effects can be observed in children and adolescents, with negative effects mainly on the psychological domain (10).

In patients aged 8–18 years with CLP, a cross sectional study on the perception of current and retrospective quality of life before treatment, including surgery and orthodontics, showed improvement in the physical, psychological, and social health domains; with the largest effects on physical function and communication (8). Still, prospective studies focusing on the oral health-related quality of life in children who have waited for delayed alveolar bone grafting and hard palatal closure are scarce. Compared to early hard palate closure, delayed repair can have an impact on speech development (11), including phonetic and phonologic disorders (12, 13) and language disruption (14), with effects on school performance (15, 16). Additionally, at the age of mixed dentition, malocclusion by its own can decrease the oral health-related quality of life (17).

The current study was performed in children with not syndromic cleft lip/ palate (nsCLP) with intentionally unrepaired nasoalveolar fistula, who have waited until the time of mixed dentition (before canine eruption) for alveolar bone grafting combined with hard palatal closure. The aim of the study was to assess the difference in the oral health-related quality of life before and 6 months after alveolar and hard palate reconstruction, in comparison to age matched children with no birth abnormalities, considering dental occlusion.

## 2 Material and methods

After the study protocol was authorized by the institutional Research and Ethics Committees (R-2019-3603-072, 21/10/2019), written informed consent was obtained from both the participants and their guardians, with the freedom to ask any questions before signing the consent form or at any time during or after the study, and the explicit right to stop participation at any time without prejudice. Data collection was conducted from August 2021 to July 2023.

### 2.1 Participants

A total of 52 children participated in the study, they were:

- 26 consecutive children (mean age ± standard deviation 8.8 ± 0.7 years; 10 girls/ 16 boys) with complete lip/ palate cleft with intentionally unrepaired nasoalveolar fistula and no other birth abnormalities [according to the International Classification of Diseases, 2019 (18)] (participants with nsCLP). They were invited to participate at the cleft lip/palate clinic of an institutional tertiary hospital for children. Seventeen (65.4%) children had unilateral cleft, and 9 (34.6%) children had bilateral clefts. They had soft tissue reconstruction at age 3–9 months (20% cheiloplasty/ 80% naso-cheiloplasty) and palatoplasty at age 14–30 months (100% two-flap palatoplasty). All of them received transverse and anterior-posterior expansion at *circa* 6 years old, and only one did not receive orthopaedic treatment. During this study, all underwent secondary bone grafting by cancellous bone from the iliac crest using Boyne technique (19), without complications. The mean fistula width was 9.3 ± 4.0 mm for all participants; 5.1 ± 2.3 mm right and 7.1 ± 3.2 mm left when it was unilateral, and 12.8 ± 3.4 mm in total when it was bilateral.
- 26 Children (mean age 9.1 ± 0.8 years, 13 girls/13 boys) with no birth abnormalities (participants without nsCLP), who were siblings or companions of patients attending the clinic

A sample size of 26 patients was estimated for covariance analysis, to find an effect size of R^2^ = 0.2 for one independent variable and 0.2 for three controlled variables, setting alpha at 0.05 and beta at 0.2 (20). This sample size included the sample size of 21 participants per group that was estimated to find a 20% difference in dental occlusion (Angle Class I) between participants without nsCLP (50%) (21) and those with nsCLP, setting alpha at 0.05 and beta at 0.2.

None of the participants in the two groups had primary otolaryngology/ neurology/ psychomotor disease, severe tooth decay or any systemic disease that could interfere with the study protocol, which were corroborated by the institutional clinical records and clinical evaluation.

### 2.2 Procedures

In all the participants, dental occlusion was assessed at inclusion in the study by the Angle classification (22, 23) and Dental Aesthetic Index (24); while the GOSLON yardstick index (25) was used just in participants with nsCLP. In all the participants, quality of life related to oral health was evaluated by the Child Perceptions Questionnaire (CPQ_8-10_) (26) to address the frequency of events over the 4 previous weeks, at two times: before and 6 months after the surgery in the group with nsCLP, and, with 6 months in-between in the group without nsCLP.

### 2.3 Assessment tools

Assessment tools were administered by a qualified specialist, who was previously calibrated (in children with no birth abnormalities; Kappa = 0.80, *p* < 0.05).

- The classification of Angle (22, 23), which comprises 3 categories with reference on the relationship between the first permanent maxillary and mandibular molar, with three categories of malocclusion.
- The Dental Aesthetic Index (DAI) (24), which comprises 10 variables of dentofacial anomalies related to both clinical and aesthetic characteristics: missing anterior teeth, incisal segment spacing and midline diastema, incisal segment crowding, largest anterior irregularity in the maxilla, largest anterior irregularity in the mandible, anterior maxillary overjet, anterior mandibular overjet, anterior open bite, and anteroposterior molar relation. To separate 4 categories of malocclusion, a total score is calculated by an equation; the categories of increasing severity are: 0. DAI ≤ 25, normal or minor malocclusion; 1. DAI 26–30, definite malocclusion; 2. DAI 31–35, severe malocclusion; 3. DAI ≥36, very severe malocclusion, probable orthognatic surgery.
- The GOSLON (Great Ormond Street London and Oslo, Norway) yardstick index (25) to assess malocclusion on the casts of participants with nsCLP by the anteroposterior arch relationship, the vertical labial segment relationship and the transverse relationship. The degree of horizontal discrepancy is measured by the overjet and the final score can be considered to be a reflection of the maxillary growth: 1= Excellent, 2= Good, 3= Fair, 4= Poor, 5= Very poor.
- The Child Perceptions Questionnaire (CPQ_8-10_) (26), which comprises 25 items categorized into 4 domains: oral symptoms (five items); functional limitations (five items); emotional well-being (five items); and social well-being (10 items). Each item has five response options: never = 0, once or twice = 1, sometimes = 2, often = 3, every day or almost every day = 4. A total score is calculated by summing all the item scores, from 0 (no impact) to 100 (greatest impact). The questionnaire also contains four introductory questions, 2 on gender and age, and 2 concerning oral health and the extent to which the orofacial condition affects the overall wellbeing. The Cronbach's alpha coefficient in a similar age sample was 0.89 (27).

### 2.4 Statistical analysis

After assessing data distribution using Shapiro–Wilks test, bivariate analysis was performed according to data distribution using either “t” test or Median test or *X*^2^, and ANOVA or Kruskal Wallis test. Then repeated measures multivariate analysis of covariance was performed with a significance level of 0.05.

## 3 Results

### 3.1 Bivariate analysis

#### 3.1.1 Dental occlusion

In participants with nsCLP, Angle classification was Class I. in 11 children (42.3%, 95% C.I. 23.3%–61.3%) and Class III. in the remaining 15 children (57.7%, 95% C.I. 38.7%–76.7%). In participants without nsCLP, it was Class I. in 17 children (65.4%, 95% C.I. 47.1%–83.7%), Class II. in 3 children (11.5%, 95% C.I.0–26.7%) and Class III. in 6 participants (23.1%, 95% C.I.6.9%–39.3%). Comparison between the groups showed no significant difference in the total score (Median test, *X*^2^ = 2.78, *p* = 0.09); however, the proportion of participants with Angle Class III. was significantly higher among participants with nsCLP than in those without nsCLP (*X*^2^.24, *p* = 0.002).

In participants with nsCLP, the DAI category was 0. in 5 children (19.2%, 95% C.I. 4.0%–34.3%), 1. in 2 children (7.6%, 95% C.I. 0%–17.8%), 2. in 4 children (15.4%, 95% C.I. 1.5%–29.2%) and 3. in 15 children (57.7%, 95% C.I. 38.7%–76.7%). In participants without CLP, the category was 0. in 14 children (46%, 95% C.I. 0%–17.7%), 1. in 4 children (15.4%, 95% C.I. 1.5%–29.2%), 2. in 15 children (57.7%, 95% C.I. 38.7%–76.7%) and 3. in 2 children (7.6%, 95% C.I. 0%–17.8%). Comparison between the groups showed significant differences on the total score (Median test, *X*^2^ = 13.01, *p* = 0.0003) and on the proportion of participants in category 3. (*X*^2^ 14.55, *p* = 0001).

In participants with nsCLP, the GOSLON yardstick index category was 1. in 11 children (42.3%, 95% C.I. 23.3%–61.3%), 2. in 6 children (23.1%, 95% C.I. 6.9%–29.3%), 3. in 8 children (30.7%, 95% C.I. 12.9%–48.3%) and 4. in one child.

**Figure 1:**
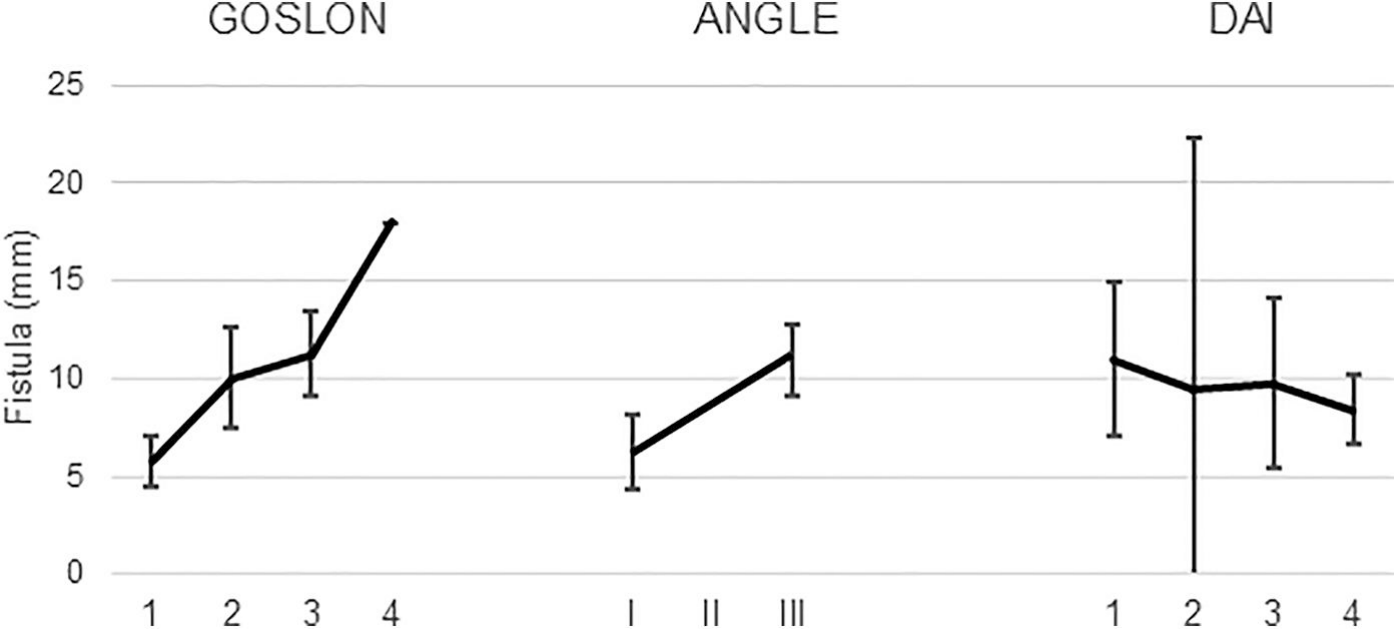
Mean and 95% confidence interval of the mean size of the fistula according to the classification provided by each of the three classifications of dental occlusion that were used to evaluate 26 children with nsCLP and intentionally unrepaired nasoalveolar fistula.

In participants with nsCLP, a larger fistula was observed according to the severity of malocclusion when it was assessed by either the Angle classification or the GOSLON yardstick index (ANOVA, F 9.99 and 9.16, *p* < 0.005), but not when it was assessed by the DAI index (ANOVA, F 0.47, *p* = 0.70) (Figure 1).

##### 3.1.1.1 Oral health -related quality of life

In the first evaluation, before surgery, participants with nsCLP with intentionally unrepaired nasoalveolar fistula showed higher scores (lower quality of life) than participants without nsCLP. The median scores on each domain were from 5 to 8.5 in children with nsCLP, with a total score of 28 (Q1-Q3 15–35); while the median scores on each domain were from 1 to 2 in children without nsCLP, with a total score of 8.5 (Q1-Q3 7–11). Six months later (after surgical reconstruction), the domain subscores and total score of children with nsCLP with fistula improved (domain scores from 2 to 3; total score 12.5, Q1-Q3 8–17), while those of children without nsCLP remained almost the same (domain scores from 1 to 3.5, total score 9, Q1-Q3 7–13), with difference between the groups (repeated measures ANOVA, F 17.06, *p* = 0.0001).

### 3.2 Multivariate analysis

#### 3.2.1 Oral health -related quality of life by group

The multivariate analysis of covariance showed the expected effect of the group on the variance of the total score and domain subscores of the C_–10_, with influence from the age at the first evaluation (beta value 0.28, 95% C.I. −0.45 to −0.10), and on the difference between the two evaluations (Table 1), with an adjusted R^2^ decrease from 0.62 to 0.19 (Table 1). The analysis by domain showed that the age contributed to the variance just on the domains of emotional well-being and social well-being, while gender contribution was observed just on the domain of functional limitations (Table 1).

**Table 1:** Adjusted R^2^, R, *p* values of the variables contributing to the variance on CPQ_8-10_ total score and subscores.

	Total score	Oral symptoms	Functional limitations	Emotional well-being	Social well-being
Adjusted R^2^ (R), *p* value	0.62 (0.81)	0.27 (0.57)	0.52 (0.75)	0.48 (0.72)	0.46 (0.71)
At inclusion in the study	<0.000001	0.0006*	<0.000001*	<0.000001*	<0.000001*
Adjusted R^2^ (R), *p* value	0.19 (0.44)	0.01 (0.30)	ns		0.24 (0.55)
At 6 months follow-up	0.03	0.30		ns	0.001*
Variables	*p* (F value)	*p* (F value)	*p* (F value)	*p* (F value)	*p* (F value)
Intercept	0.0001 (16.98)*	0.41 (0.68)	0.005 (8.63)*	0.002 (10.38)*	0.001 (12.16)*
Age	0.08 (3.189)	0.68 (0.17)	0.09 (2.87)	0.030 (5.00)*	0.043 (4.31)*
Gender	0.21 (1.55)	0.15 (2.12)	0.013 (6.55)*	0.90 (0.01)	0.79 (0.06)
Group	<0.000001 (82.31)*	0.0004 (14.29)*	<0.000001 (24.52)*	<0.000001 (29.86)*	<0.000001 (60.16)*
Gender and Group	0.055 (3.84)	0.85 (0.03)	0.004 (8.95)*	0.43 (0.61)	0.72 (0.12)
Repeated measures (R1)	0.001 (11.78)*	0.57 (0.31)	0.08 (3.04)	0.002 (10.00)*	0.009 (7.31)*
R1* age	0.003 (9.43)*	0.70 (0.13)	0.16 (2.01)	0.007 (7.88)*	0.025 (5.34)*
R1 * gender	0.78 (0.07)	0.71 (0.13)	0.15 (2.03)	0.67 (0.17)	0.067 (0.06)
R1 * group	0.0001 (17.08)*	0.006 (8.04)*	0.002 (10.69)*	0.001 (12.01)*	0.001 (11.50)*
R1 * Gender * Group	0.78 (0.07)	0.96 (0.002)	0.09 (2.84)	0.67 (0.17)	0.084 (3.10)

Significant relationships are highlighted using *. At inclusion in the study the variables that were included in the analysis explained 62% of the variance of the perceived oral health-related quality of life; six months later (after surgical reconstruction in children with nsCLP), this percentage decreased to 19%. The repeated measures (R1) results denote differences between children with/without nsCLP in all the domains and the total score; since participants with nsCLP reported changes on quality of life while those without nsCLP reported no change.

#### 3.2.2 Oral health -related quality of life by dental occlusion

Multivariate analysis of covariance on the contribution of the dental occlusion to the oral health-related quality of life (Table 2) showed that, in all the participants, either the Angle classification or the DAI Index contributed to the variance on the oral health-related quality of life at the time of inclusion in the study, as well as on the difference between the two evaluations, with a contribution from age, but not from gender (*p* < 0.05). In the participants with nsCLP, dental occlusion assessed by the GOLSON yardstick index contributed to the variance on the difference between the two evaluations, with influence from age and dental occlusion, and interaction between these two variables (Table 3). This contribution was evident on the emotional well-being and social well-being domains. Gender influence could not be evaluated due to the data distribution among the categories.

**Table 2:** Adjusted R^2^, R and *p* values of the variables contributing to the variance on CPQ_8−10_ total score of all participants, according to the dental occlusion evaluation by the Angle classification and the DAI index.

	Angle	DAI index
Adjusted R^2^ (R)	0.21 (0.55)	0.33 (0.61)
*p* value	0.008	0.00004
Variables	*p* (F value)	*p* (F value)
Intercept	0.001 (11.87)*	0.0009 (12.33)*
Age	0.029 (5.07)*	0.008 (7.57)*
Gender	0.34 (0.91)	0.48 (0.48)
Dental occlusion	0.045 (3.30)*	0.00007 (18.64)*
Repeated measures (R1)	0.0004 (14.37)*	0.0007 (12.85)*
R1* Age	0.0008 (12.70)*	0.0004 (13.94)*
R1* Gender	0.86 (0.02)	0.78 (0.077)
R1* Dental occlusion	0.069 (2.82)	0.024 (5.41)*

Significant relationships are highlighted using *. Age and dental occlusion contributed to more than one fifth of the variance of the total score on the perceived oral health-related quality of life. The results suggest that the DAI Index could be a better tool for the follow-up of the dental occlusion contribution to the perceived the perceived oral health-related quality of life.

**Table 3:** Variables contributing to the variance on the total score and subscores on the CPQ_8−10_ in participants with nsCLP according to the dental occlusion evaluation by the GOSLON yardstick index.

	Total score	Oral symptoms	Functional limitations	Emotional well-being	Social well-being
Adjusted R^2^ (R), *p* value	0.55 (0.79)	Ns	ns	0.32 (0.66)	0.49 (0.76)
At inclusion in the study	0.0003*			0.017*	0.001*
Adjusted R^2^ (R), *p* value	0.55 (0.79)	Ns	ns	0.61 (0.82)	0.35 (0.67)
At 6 months follow-up	0.0003*			0.000009*	0.011*
Variables					
Intercept	0.001 (14.37)*	0.12 (2.58)	0.24 (1.40)	0.016 (6.91)*	0.006 (9.28)*
Age	0.093 (3.10)	0.49 (0.48)	0.67 (0.18)	0.132 (2.46)	0.095 (3.05)
Dental occlusion	0.83 (0.28)	0.48 (0.85)	0.66 (0.53)	0.26 (1.42)	0.33 (1.19)
Repeated measures (R1)	0.0003 (18.25)*	0.89 (0.017)	0.12 (2.49)	0.002 (12.35)*	0.028 (5.61)*
R1* age	0.0007 (15.89)*	0.95 (0.003)	0.21 (1.61)	0.002 (11.58)*	0.050 (4.31)
R1 * dental occlusion	0.0001 (11.70)*	0.33 (1.20)	0.84 (0.27)	0.001 (7.61)*	0.0005 (9.05)*

Significant relationships are highlighted using *. At the two times of evaluation, both the age and the dental occlusion contributed to 55% of the variance of the total score on the perceived oral health-related quality of life; however, no significant (ns) contribution was observed on “oral symptoms” and “functional limitations”. The age and dental occlusion also contributed to the difference between the two evaluations, with an interaction on two dimensions, “oral symptoms” and “functional limitations”.

## 4 Discussion

The current study was aimed to assess the difference in the oral health-related quality of life before and 6 months after alveolar and hard palate reconstruction in children 8–10 years old, in comparison to age matched children with no birth abnormalities, considering dental occlusion. The oral-health related quality of life (either with or without nsCLP) was found to be significantly related to dental occlusion and to age, with interaction between these two variables on the improvement observed after the surgical reconstruction, mainly on the emotional and social well-being domains of the CPQ_8-10_.

The general results of the current study are consistent with a systematic review and meta-analysis on oral-health related quality of life in children and adolescents with CLP, aged 8–19 years old. The meta-analysis of 14 studies, comprising 1,185 patients with CLP and 1,558 healthy controls. showed that oral health-related quality of life was slightly decreased in those with CLP, particularly on the functional, emotional and social domains (28).

The main physical consequences of CLP are facial and functional impairments. However, children with unrepaired cleft palate at school age, waiting for alveolar bone grafting, face challenges that extend beyond their physical health and functioning. This study shows that the most evident effects of late reconstruction could be on the emotional and social domains. This result is consistent with the evidence that, compared to healthy children, children with CLP may experience increased emotional disarrays and greater difficulties in social interactions (29).

Adequate evidence to support any specific chronologic age for bone grafting is lacking (30, 31). However, on the psychosocial effects of cleft lip and palate, some differences have been found according to the type of cleft (32). A recent consensus recommends preliminary screening with additional diagnostic tests/ treatments according to the screening results (33). The findings of this study emphasize the need of early guardian and child counselling, with clear explanation on the risk/benefit balance of the specific therapeutic options. Always taking into account the physical/ mental/ sociocultural characteristics and needs of each patient, along with the family effort and the access or not to long term multidisciplinary health care.

Although 8–10 years of age could seem a narrow period, the influence of the age was noticeable on both emotional well-being and social well-being. Due to their ongoing development, the structure of children's self-concept and perception of health is age dependent. In early childhood, the emerging abilities allow positive interactions with peers and adults (34). At the age of mixed dentition, children begin elementary school, which has influence on their social and emotional development (35). The first years at school are concerned with the acquisition of new social roles. At that time, children are influenced by the interaction with both the school personnel and their peers; their performance is related to their ability to get along with them, and to follow the rules of the environment (36). As children are exposed to other children, they become increasingly more aware and introspective, their self-consciousness can lead to feelings of embarrassment, which could be provoked by their appearance or malfunction. The understanding of social categories contributes to organize their behaviours and develop their character (37).

In this study the effect of dental occlusion disarrays on oral health related quality of life was observed in the two groups of participants, with/without nsCLP. The results are consistent with the evidence showing that malocclusion can decrease the oral health-related quality of life at the age of mixed dentition (17). The analysis also showed that both the Angle's classification and the DAI Index can be useful to evaluate the contribution of malocclusion to health related quality of life in children aged 8–10 years, but more consistent results were observed on the DAI Index (Table 2). In contrast, the Angle's classification had significant correlation with the size of the fistula, while the DAI Index had no correlation. These results are congruent with the properties of each tool. The Angle's classification mainly depends on the assessment of molar relation, which is a structural feature (22); while the DAI Index includes both occlusal and aesthetics aspects, accounting for dentition, space and occlusion (24). In participants with nsCLP, assessment by the GOSLON yardstick index was consistent with the results of international studies (38). Additionally, this study showed that the severity of malocclusion, at the critical age of mixed dentition, can influence the difference perceived on the oral health-related quality of life after surgical reconstruction, particularly on psychosocial aspects.

The main limitation of the current study is the small sample size, which allowed to assess just the most evident correlations without denying other potential relationships; although, it allowed focus in some specific issues, such as the influence of age and the main affected domains of quality of life. The nature of the malformation precluded blinding, while similar sociocultural background of the participants with/without nsCLP allowed comparisons between the study groups within the same context. These two factors pose a limit on the generalizability of the results. Additionally, the design of the study cannot disentangle the effects on the quality of life attributable to the surgical reconstruction from those derived from the multidisciplinary care required by patients with CLP; and a more extensive follow-up would be required to recognize the multidimensional effects of the surgical reconstruction. Another limitation to ponder is the absence of children with full reconstruction of the palate at an early age, which was out of the scope of the study.

The results support the relevance of early evaluation of dental occlusion in both children with/without nsCLP, to start prompt orthopaedic treatment. In children with nsCLP with intentionally unrepaired nasoalveolar fistula, the earliest possible reconstruction could be beneficial for their perceived emotional and social well-being.

## Data Availability

The raw data supporting the conclusions of this article will be made available by the authors, without undue reservation.
